# Rapid surveys on violence against women in crisis contexts: decision-making guidance based on the UN Women Rapid Gender Assessment surveys on violence against women during COVID-19

**DOI:** 10.1016/S2214-109X(24)00278-X

**Published:** 2024-10-16

**Authors:** Raphaëlle Rafin, Nabamallika Dehingia, Juncal Plazaola-Castaño, Anita Raj

**Affiliations:** a UN Women, New York, NY, USA; b Center on Gender Equity and Health, University of California, San Diego, CA, USA; c Newcomb Institute, Tulane University, New Orleans, LA, USA; d Tulane School of Public Health and Tropical Medicine, New Orleans, LA, USA

## Abstract

Rapid surveys or assessments offer the possibility to collect data in contexts where classic data collection is not feasible (such as health, humanitarian, or climate crises) and when evidence-based urgent action is needed to mitigate the effects of the crisis. Until the past 5 years, rapid surveys were not widely used by practitioners, researchers, or policy makers to measure the effect of crises on violence against women due to a paucity of empirical evidence on their safety and likely utility in such contexts. In recent years, and particularly during the COVID-19 global pandemic, UN Women led the piloting and implementation of such surveys in various countries. We use our experiences from this work and other studies to offer concrete decision-making guidance—in the form of a checklist—for whether to conduct rapid surveys on violence against women in crisis contexts, with consideration of their value, risks, and the minimum safeguards needed to implement this type of work.

## Introduction

Globally, more than one in four women aged 15 years and older have experienced physical or sexual violence, or both, by an intimate partner in their lifetime, and one in 17 have experienced non-partner sexual violence.^1^, ^2^ 15–99% of women in low-income and middle-income countries report sexual harassment experiences.^3^ These rates are unacceptably high, but research suggests decreasing trends in some national contexts over the past 30 years, at least in the case of intimate partner violence (IPV).^4^ Declines are probably a consequence of implementation of prevention programmes, and implementation of legislation and policies in many countries to end violence against women (VAW).^5^ Nonetheless, the existing prevalence does show that we are still a long way from achieving the UN Sustainable Development Goal (SDG)^5^ to end VAW globally. Furthermore, in situations of crisis, gains made in VAW prevention can be lost.

Extensive research has documented increases in VAW in situations of conflict, and even use of sexual violence against women as a means of war, and growing evidence similarly shows increased risk for VAW in disaster settings.^6^, ^7^ IPV increased among women and girls who were residents of areas of Louisiana and Mississippi affected by Hurricane Katrina, with higher odds of an increase for those whose homes endured greater damage and those who remain displaced.^8^, ^9^, ^10^ Escalation of sexual violence and exploitation of girls subsequent to Ebola outbreaks was documented in west Africa, and exacerbated in contexts where conflict followed outbreaks.^11^ Many, but not all, countries saw an increase in IPV during the COVID-19 pandemic.^12^ Across studies, risk of IPV was greater for some groups that were more vulnerable, such as women who are displaced and those already socially or economically marginalised. Every crisis context is unique in terms of its effect on women's experiences of violence and who might be most affected, with each crisis warranting a rapid investigation of VAW for timely and effective policy responses.

## Should rapid surveys on VAW be conducted in crisis contexts?

Despite the recognised value of data on VAW to guide rapid responses in crisis, the value of rapid surveys in crisis situations is disputed, because response to the emergency itself has to be the greatest priority.^13^ Collecting data from women and girls in crisis contexts, particularly if this requires them being interviewed about their experiences of violence through remote surveys, where privacy can be more difficult to ensure, could pose risk of increased violence or further trauma.^14^ Following specific safety and ethical standards can help, but even beyond this, the decision to conduct rapid surveys should be a thoughtful and critical process. Any existing data that can provide relevant insights should be thoroughly investigated; new data should be collected in ways that ensure minimal or no risk of harm and maximum societal benefit.

In most cases, data are necessary to make the case for the need to invest in and address the increased risks of violence for women and girls in times of crisis. The data obtained through rapid surveys can also add to the larger literature, which, when aggregated, can contribute to the understanding of future risks and consequences of crises on women and girls. To decide whether or not to undertake rapid surveys, and how to conduct them, in crisis contexts is thus paramount. In this Viewpoint, we present key learnings from the UN Women's Rapid Gender Assessment survey on VAW during the COVID-19 pandemic, implemented in 2021 across 13 countries, which used remote telephone-based surveys.^15^ Based on these learnings and the process of decision making adopted during the survey design, we present a checklist of six steps that researchers and practitioners should consider before and while conducting rapid surveys on VAW in crisis contexts, including pertinent points to consider to ensure that any work during this time of crisis is both effective and ethical.

## Learnings from experiences with rapid surveys on VAW during the COVID-19 pandemic

### Step 1: assess need for data

Early in the pandemic, domestic violence and sexual assault services in many national contexts began receiving an increase in phone calls and digital outreach, suggesting an increase in VAW, but there were insufficient data to confirm an increase in IPV and guide responses for service providers working in areas affected by COVID-19.

Before deciding whether the collection of new data warrants the conduct of rapid surveys, it is imperative to first look at different existing data sources (eg, surveys, administrative records, and qualitative studies) as these might already have evidence capable of informing policy response. When planning the UN Women's Rapid Gender Assessment survey, no large-scale data collection efforts could be identified to understand VAW needs in the countries of focus. It is also important to ensure that other stakeholders are not currently collecting data on VAW that can be used. Again, we identified no large-scale, population-based study or survey being done in the targeted countries.

### Step 2: confirm value added of the survey at the local level

Another key aspect to consider when deciding whether to launch a rapid survey on VAW is the usefulness of such data in the local context. In many situations, rapid surveys of women's self-reported experiences of violence might not be the best option (eg, in contexts of intense migration, in which not enough women can be reached via telephone surveys to make useful estimates, or where ensuring privacy during telephone calls might be impossible). In such cases, other options such as interviews with service providers, or examination of data from service records, could be explored. New, large-scale, survey data collection that is designed to be population-representative might not be useful if VAW data cannot be collected directly from women and girls in confidential and safe settings, or over a sufficiently short enough period to capture the given population's immediate needs. Telephone surveys conducted at scale could be done during the COVID-19 pandemic because, at a certain point, migration had declined and infrastructure to enable calls were in place.

### Step 3: establish feasibility in terms of adequate representativeness

For robust population and subpopulation estimates of VAW, adequate representation of different groups of women and girls must be ensured, particularly those more at risk of violence. However, remote interview techniques, such as telephone surveys (as used for the UN Women Rapid Gender Assessment survey on VAW during COVID-19), pose unique challenges for representation as mobile phone access is not universal, especially in low-income and middle-income countries. Supplemental survey sampling to ensure inclusion of under-represented demographic groups is necessary. Researchers need to consider how to maximise representativeness and inclusivity to meet local VAW prevention needs. The UN Women Rapid Gender Assessment survey on VAW during COVID-19 set country-specific quotas on the basis of national official figures for total number of sampled respondents based on age groups and region. Additional samples were also used for groups that were harder to reach, such as older women (≥60 years) and supplemental databases (eg, previous respondents in other surveys who indicated they could be contacted for future similar activities, but still restricted to those who own mobile phones). Geographical targets were relaxed in favour of meeting demographic targets when required, to collect data that were more representative demographically than geographically. Although these modifications are less than ideal for large-scale surveys to provide population estimates, there must sometimes be some compromises to ensure data are collected.

### Step 4: ensure safety of respondents

Previous evidence, standard ethical practice for surveys on VAW, and expert input were used to guide the approach for the UN Women Rapid Gender Assessment survey on VAW during COVID-19. An expert technical advisory board of 11 members was created comprising VAW researchers and practitioners who work across national settings and crisis contexts.

Existing gold-standard ethics protocols for survey research on VAW were followed, which include a standard approach recognised and used by survey research groups focused on VAW research at scale, including those within the UN and WHO;^16^, ^17^, ^18^ these protocols were adapted for telephone surveys. The protocol included community engagement and local partnerships, careful selection and training of interviewers, informed consent with confirmed confidentiality and privacy for participants, clarity that participants can decline surveys or stop interviews without penalty, confirmation of a safe word if the participant is concerned for their safety or if confidentiality is breached, VAW referrals for participants, and psychosocial support for interviewers (panel).

Panel **Protocol for safe and ethical data collection on violence against women**

**Partner with local experts:**

• Some forms of violence against women (VAW) are socially and culturally normative, and contextual considerations are needed to undertake research on VAW. Engagement with local experts, from within research and academia but also within local communities and VAW community organisations, can strengthen survey development, generation of locally meaningful research questions, and dissemination and use of data in nations and communities.

**Carefully select and train interviewers:**

• We recommend gender-matched interviewers who are not from the same or neighbouring communities as the respondent. Interviewers should be trained on survey interview methods, gender-based violence and referrals, non-judgemental but warm engagement and responses, how to recognise and respond to distress, and how to recognise and respond to situations in which the interviewee is not alone or could be overheard. Include scripted statements and responses in survey protocols when possible.

**Provide survey introduction and informed consent:**

• Introduce the survey as a survey on women's issues or women's health, rather than on VAW. Obtain informed consent before survey administration with protocols that have received institutional review board approval. Confirm that the respondent can refuse to answer a question or cease the interview at any time with no penalty.

**Ensure confidentiality:**

• Include no personally identifiable information of the respondent on the survey. If there is a need to have this information connected to the data, use a unique identifier on the survey that can be connected to separately filed identifiable information. Inform the respondent that their information will be kept confidential, and ask them not to share with others the questions they have been asked. Store de-identified survey data securely.

**Confirm privacy for all interviews:**

• Both the interviewer and the respondents should be in a private setting at the time of the interview. Before starting the interview, confirm with the respondent that they are alone (excluding a child younger than 2 years) and, once confirmed, construct a plan for them to indicate when they are no longer alone or if someone is listening on the line (eg, a safe word). Confirm that speakerphone is turned off. If an online interview is being conducted, confirm that a digital privacy shield can hide the survey.

**Provide accessible VAW referrals to all participants:**

• Ensure you have a list of domestic violence service agency referrals that can be accessible to the participants locally and via helplines. Make sure the list is up to date. Provide to all women regardless of their responses to the survey, and note that the referrals are for them and that they can share them with anyone they know confidentially.

**Ensure access to emotional support for interviewers:**

• Surveys on VAW can be difficult for interviewers as well as respondents. Provide regular monitoring and check-ins with interviewers, and assess their wellbeing periodically. Have no-cost psychosocial support available to interviewers on an as-needed and as-requested basis.

Field observations are also important. For the UN Women Rapid Gender Assessment study,^15^ 10% of all interviews were monitored by fieldwork supervisors to identify any systemic issues with interviewers (eg, high percentage of short interviews). Any interviewers who were identified underwent corrective training steps (eg, rebriefing or additional training), and data from those interviews with issues were not included in the analysis. No interviews were recorded to reduce risk for breach of confidentiality.

Survey data were monitored and tracked via weekly data reviews. Data validation was conducted on a continuous basis throughout fieldwork by the survey management team, to ensure that the collected data were of high quality. Data verification and related quality assurance processes ensured that the special ethical and safety protocols for the survey were being appropriately followed. Regarding data collection, no concerns were found related to risk to participants or to breach of confidentiality, nor were there any severe adverse events (eg, a resultant incidence of violence due to survey participation) related to this work, suggesting that these surveys can be done safely.

Over the course of the interview, respondents were repeatedly asked to confirm that they were in a quiet, private place, where no one older than 2 years was able to listen to the interview, and respondents were instructed that they should not be on speakerphone. Less than 2% of respondents across all countries could not maintain privacy for these interviews and, in these cases, interviews were stopped.^19^ There were no study dropouts and no respondents declined to answer any questions, including those relating to direct VAW. A safe word was provided to allow participants to signal the need to end the interview; its use was seen in less than 1–4% of participants per country.^19^ No concerns were identified during data monitoring with the quality of the data. The number of respondents who completed the survey was high, with only a 15% dropout rate across all 13 countries.^19^

### Step 5: clarify measures for VAW

Careful consideration was given as to whether data on VAW could effectively be collected via telephone-based rapid surveys during COVID-19, as the best existing evidence on VAW is from household surveys with a high number of questions to assess VAW comprehensively. Questions that were more brief were required for the telephone survey, selected from short measures (eg, dichotomous and Likert scale questions) and with expert input; novel indirect measures of VAW (ie, not asking directly about an individual's experience with IPV) were also validated due to concerns from some experts regarding respondents’ safety with direct questions. For these indirect measures, survey questions (about self-experience or knowledge of others’ experience of VAW), vignettes, and list randomisation were used.^20^, ^21^ Brief direct VAW measures were included in one country. Before scaling up direct survey measures, cognitive interviews were done by trained research staff, and these participants reported that the direct measures were clear and non-problematic (indirect measures were also tested to ensure they were understood by respondents).^19^

No reports were received of adverse events (eg, family conflict or incident of violence) during the survey, nor did any respondents decline to respond to the direct question about their individual experience of VAW. Respondents’ reports (direct and indirect) were analysed, and all VAW questions proved valid on the basis of their strong inter-correlations with each other. The UN Women's survey also included cognitive testing of the questions relating to VAW; respondents indicated that the direct question was understandable, acceptable, and socially valuable with regard to increasing social awareness of an issue that affects so many women.

Regardless of capacities for undertaking survey research, there is no value in conducting a survey if you are not clear on what you want to measure, why you want to measure it, and how the data you collect can and will be used to improve human health, development, or security. In the UN Women's survey, questions regarding direct experience of VAW by the respondent were all answered (ie, no missing data). Data from these direct questions are the most impactful in terms of aligning with SDG targets to reduce VAW, and are the most easily interpreted by stakeholders and the wider public. Therefore, we recommend that brief, but direct questions on experiences of VAW are included in rapid surveys conducted during times of crises, provided aforementioned ethical and safety measures are ensured.

### Step 6: plan data dissemination and use

Ensuring use of the data by local policy makers and advocates is one of the necessary requirements of any data collection exercise. This data use is particularly true for crisis situations, as the benefits of any action in this context must outweigh any potential additional risk induced by the survey. In the UN Women Rapid Gender Assessment survey on VAW during COVID-19, partnerships and protocols for use of the collected data (by national institutions and civil society organisations) were set up before launching the survey to help guarantee national ownership and use of data. UN Women, as the data producer, implemented an advocacy strategy to ensure that the data would be used in the design of VAW policies and measures during the pandemic and beyond, through regular engagement of national partners at every step of survey design, sampling approvals, field work, and dissemination of results.

Uptake of the data from this survey has already led to positive outcomes at the local level, and a year later, evidence of data use was collected from Colombia.^22^ Disaggregated microdata from one of the regions in Colombia were shared with decision makers in local institutions and authorities, and were used to raise awareness of VAW through radio and television programmes. The data were used to establish benchmarks, identify local gaps in domestic violence services and prevention activities, and adapt responses for violence prevention and survivor support that informed the local development plan.^22^ Concrete actions included the tripling of the budget allocated to gender issues in the Municipal Development Plan—from COP400 million (US$83 000) a year before the pandemic, to COP1·2 billion ($250 000) in 2021—the creation of a technological training and provision of tools on providing attention to gender-based violence survivors for the Metropolitan Police, access to a diploma in gender at the Cooperative University of Colombia for the commissioners responsible for referrals for gender-based violence, and the reopening of the Support Unit for Women Victims of Violence (which had closed during the pandemic due to administrative issues) in May, 2022, which offers legal clinics and psychological support for an average of 20 survivors of VAW per month. This example of data use from Colombia is a good illustration of how nationally produced data can directly inform local solutions.

## Conclusion

Global crises related to war, disaster, and disease continue to emerge, and in such contexts, we too often see a concurrent rise in VAW. Understanding VAW in contexts of crisis can be important to help ensure that the needs of women and girls are sufficiently considered. Rapid surveys on VAW can be crucial to such efforts, but they cannot be done without serious considerations. Response to the crisis must take precedence, and allocation of time and resources towards research must be secondary to these needs. Furthermore, this research only has value if it can swiftly provide relevant insights for applied solutions. Hence, rapid surveys on VAW are only justifiable if they can be done safely, rigorously, effectively, and when there is the capacity and political will to act quickly on these data, and provide relief and protection to women and girls. Rigorous survey methods and valid measures should also be prioritised if a survey is conducted, and these methods should be ready to address potential non-response biases, measurement errors, and sample representativeness to ensure high-quality data.^23^, ^24^

UN Women recently led a Rapid Gender Assessment survey on VAW during COVID-19 across 13 countries, and found that the data could be collected safely and the survey could yield data for programmes and policies. In this process, we found that our response to the series of questions outlined in our six-step checklist—as outlined in this Viewpoint—facilitated not only our decision making while conducting the survey, but also our plans for how to support data use. To that end, we provide a usable checklist to help set a standard in the field as to when and how to conduct as rapid survey on VAW during crisis, and what is important to ask during design and before implementation of a survey (figure). When completing the checklist, all answers to the questions must be yes to ensure that a rapid survey on VAW during a crisis adequately considers the needs of women and girls in this context and to reduce longer-term costs to nations from crises due to trauma. Rapid surveys on VAW can rapidly serve local contexts and can also offer important insights to help prevent or reduce the effects of crisis on escalation of VAW. The benefits of these data, if safely and effectively collected, can be far reaching. Public health surveillance is necessary for evidence-based prevention and control of disease and injury.^25^ Rapid surveys can effectively identify immediate needs of people affected by crisis and at risk for escalation in VAW, facilitating and even holding decision makers accountable to address the identified concerns.^26^ Furthermore, these rapid surveys can offer baseline data to assess changes over time for interventions that are put into effect to address the concerns identified.

**Figure:**
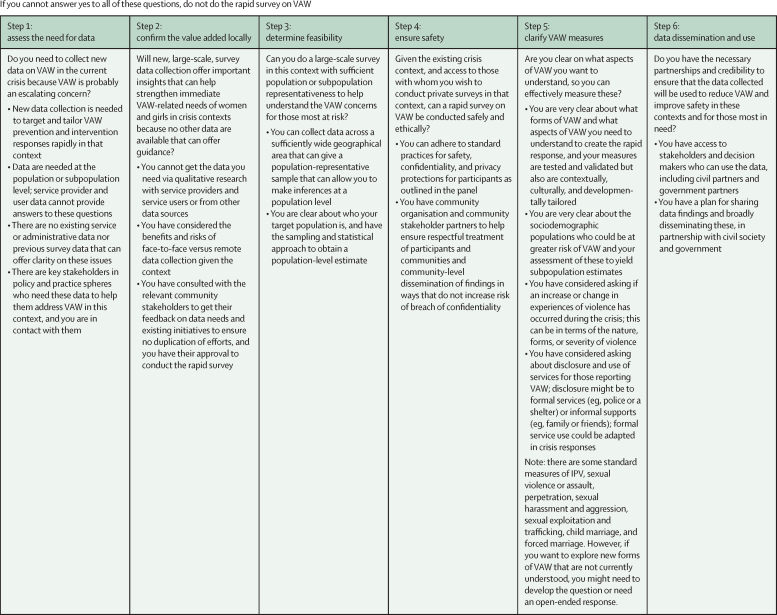
Checklist for decision making around implementing rapid surveys on VAW during times of crisis IPV=intimate partner violence. VAW=violence against women.

However, more effort is needed to maintain standard administrative data on VAW at national and subnational levels to reduce reliance on rapid survey research. Most importantly, efforts to eliminate VAW need to be a greater priority for policy makers and other stakeholders, because even in the absence of crisis, VAW remains a leading cause of morbidity and mortality for women and girls in every nation.^27^

### Contributors

## Declaration of interests

We declare no competing interests.
